# Histopathological Studies of “CH1641-Like” Scrapie Sources Versus Classical Scrapie and BSE Transmitted to Ovine Transgenic Mice (TgOvPrP4)

**DOI:** 10.1371/journal.pone.0022105

**Published:** 2011-07-13

**Authors:** Anna Bencsik, Thierry Baron

**Affiliations:** Unité Maladies Neurodégénératives, French Agency for Food, Environmental and Occupational Health Safety (ANSES), Lyon, France; University of Melbourne, Australia

## Abstract

The possibility of the agent causing bovine spongiform encephalopathy (BSE) infecting small ruminants is of serious concern for human health. Among scrapie cases, the CH1641 source in particular appears to have certain biochemical properties similar to the BSE strain. In France, several natural scrapie cases were identified as “CH1641-like” natural scrapie isolates in sheep and goats. The Tg(OvPrP4) mouse line expressing the ovine prion protein is a sensitive model for studying and identifying strains of agents responsible for scrapie and BSE. This model is also very useful when studying specific scrapie source CH1641, known to be not transmissible to wild-type mice despite the similarity of some of its biochemical properties to those of the BSE strain. As it is important to be able to fully distinguish CH1641 from BSE, we herein report the histopathological data from CH1641 scrapie transmission experiments compared to specific cases of “CH1641-like” natural scrapie isolates in sheep, murine scrapie strains and BSE. In addition to the conventional vacuolar lesion profile approach and PrP^d^ brain mappings, an innovative differential PET-blot analysis was introduced to classify the different strains of agent and revealed the first direct concordance between ways of grouping strains on the basis of PrP^d^ biochemical characteristics.

## Introduction

Transmissible spongiform encephalopathies (TSEs) are a group of fatal neurodegenerative disorders that include Creutzfeldt-Jakob disease in humans, bovine spongiform encephalopathy (BSE) in cattle, scrapie in sheep and goats and chronic wasting disease in mule deer. The neuropathological changes typical of TSEs are vacuolar damage, neuron loss, astrogliosis and abnormal deposition of the disease-specific form of the normal cellular prion protein (PrP^c^). As the disease progresses, it is admitted that PrP^c^ is converted into an abnormal protease-resistant scrapie prion protein (PrP^sc^, Sc for scrapie associated/PrP^res^, res for protease resistant), a cell-surface sialoglycoprotein with a concentrated ß-sheet conformation, that then accumulates in the diseased brain [1]. This pathological prion protein, “PrP^d^” where d stands for disease-related, is regarded as the most specific element of TSEs, also called prion diseases.

Until recently, in contrast to the diversity of experimental strains originating from scrapie, the infectious agent responsible for the disease in cattle was thought to be extremely uniform and stable even following transmission to other species, and was thus considered to belong to a single major strain of infectious agent [2], [3]. Because a relationship has been established between the variant form of Creutzfeldt-Jakob disease in humans and the BSE agent infecting cattle [4], [5], it is crucial to recognize the different types of infectious agents responsible for TSEs in order to secure public health. Biochemical tests are widely used for this purpose, particularly to distinguish scrapie from BSE cases using criteria such as migration patterns or immunoreactivity of the prion protein resistant to enzymatic digestion, PrP^res^ [6]–[12]. An immunohistochemical “peptide mapping” method has proven valuable for distinguishing strains in the brain and lymphoid tissue of natural hosts [13]. These methods have been used to investigate possible transmission of the BSE agent under natural conditions to sheep and goats [7], [8], [10], [12], [14]–[16] and, intriguingly, revealed a few cases of TSEs in sheep that showed partial similarities to experimental ovine BSE. The prototype of such cases is the CH1641 scrapie source, derived from a natural British scrapie-affected Cheviot sheep, passaged several times in sheep and goats [17] and characterized by a lower apparent molecular mass of unglycosylated PrP^res^, very close to that found in ovine BSE [7], [9], [15]. Similarly, our group previously reported seven French natural ovine “CH1641-like” isolates, compared with CH1641 and BSE [18], [19], [20]. In this study, molecular characterization using the immunohistochemical “peptide mapping” method was helpful in distinguishing these isolates—both experimental (CH1641) and natural (O100 and O104)—from BSE [20].

Strain typing studies are usually performed after transmission of the disease to various types of rodent, but another feature of CH1641 is that it is not transmissible to wild-type mice [17]. The development of transgenic mice expressing the ovine prion protein—such as the TgOvPrP4 line that expresses the ARQ allele—to detect and characterize the infectious agent involved in sheep prion diseases has been decisive for these types of isolate. The CH1641 isolate and the “CH1641-like” scrapie cases were successfully shown to be transmissible in this model as reported recently [6], [18]. Following the transmission of the disease to TgOvPrP4 mice, compared with CH1641, each of three similar French natural TSE isolates (TR316211, O100 and O104) shared some molecular similarities with ovine BSE, particularly the low apparent molecular mass of unglycosylated PrP^res^ and weak PrPres labeling by P4 monoclonal antibody [18].

The present paper aims to report the complete histopathological studies of the second passage experiments with these mice. The results of strain typing CH1641 and “CH1641-like” natural scrapie in sheep in this model are compared with those of transmission of mice scrapie strains (for which first passage data were previously reported [21]). The results of BSE transmission are also reported here as a control, knowing that from a histopathological point of view it is remarkable that once transmitted to the TgOvPrP4 mice, the BSE agent resulted in the deposition of a pathological form of PrP as florid plaques of an amyloid nature [22]–[25] whatever the original source (cattle, sheep, cheetah or goat). The presence of this specific and rare feature, typically found in vCJD, can therefore be used to distinguish the BSE agent easily and accurately in this sensitive model.

To these conventional biochemical and histopathological studies of prion strains was added the PET-blot method, which offers an easy, quick assessment of PrP^res^ mapping [26]. This study proposes an original way of learning more about the specific features of these strains by introducing an epitopic PET-blot analysis for the first time in such cases.

## Results

### Transmission of “CH1641-like” natural scrapie cases

**Figure 1:**
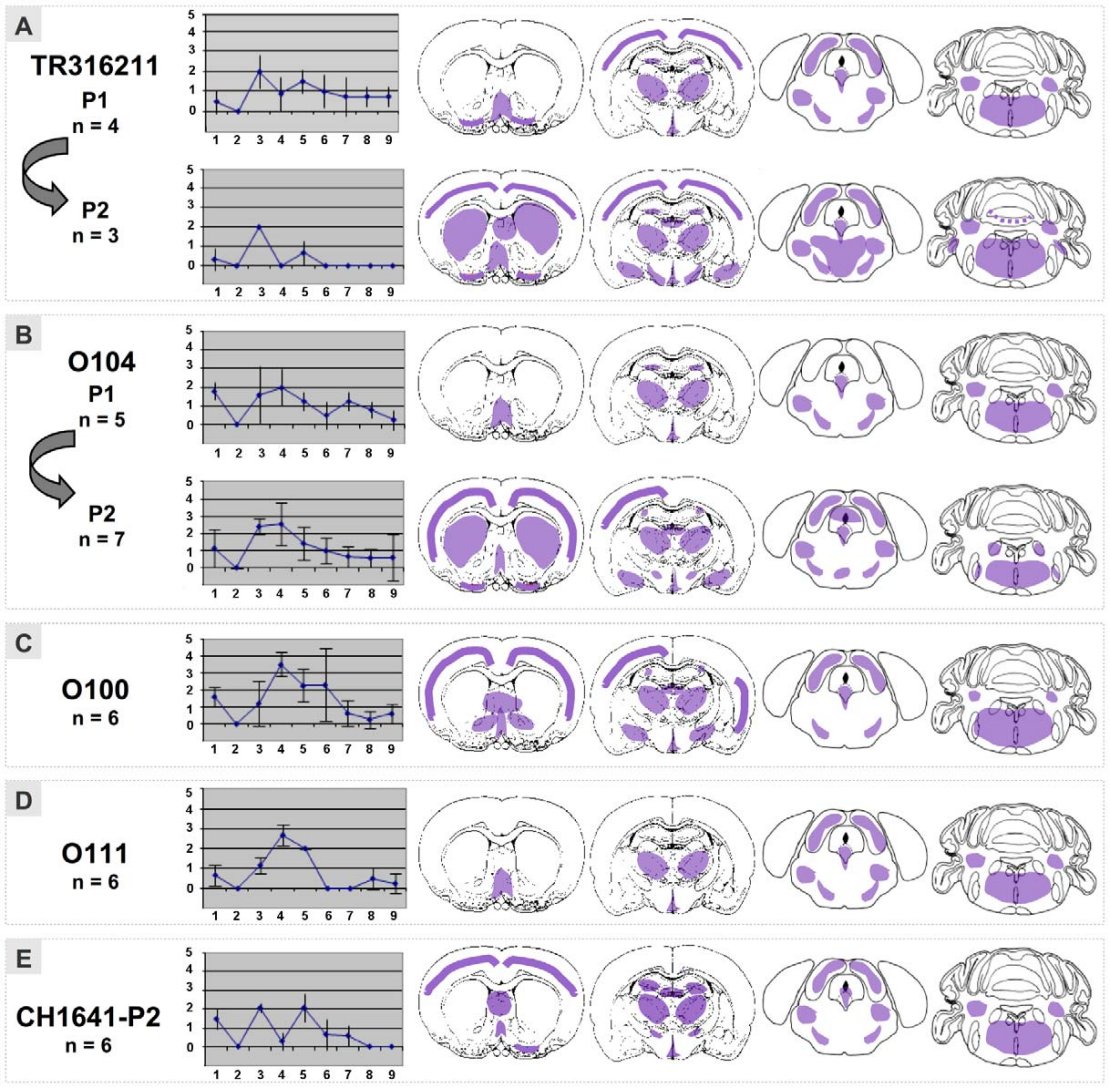
Lesion profiles and PrP^d^ mapping (schematic representation of PrP^d^ distribution in violet within 4 brain levels analyzed) observed in the brain of Tg(OvPrP4) mice (n = 3 to 7) infected with either CH1641, “CH1641-like” French scrapie isolates on the first or second passage (TR316211, O104, CH1641) or an isolate different from CH1641 (O111). 1. dorsal medulla nuclei, 2. cerebellar cortex, 3. superior colliculus, 4. hypothalamus, 5. central thalamus, 6. hippocampus, 7. lateral septal nuclei, 8. cerebral cortex at the level of thalamus, 9. cerebral cortex at the level of septal nuclei.

The new data presented here allow us to complete the study for which the biochemical data have previously been reported [6], [18]. Table 1 summarizes the survival data and attack rates. In order to facilitate interpretation of second passage data, the incubation periods and attack rates of the first passage experiments previously reported [6], [21] are also given. The mean survival periods in both first and second passage experiments appear very similar. The lesion profile of the TR316211 isolate closely resembles the lesion profile of the CH1641 transmission study. It was quite different from the O100, O104 and O111 isolates, with less vacuolation. The lesion profiles for both TR316211 and the O104 isolate, are remarkably similar when comparing first and second passage experiments (Fig. 1). However, because the lesion profile for TR316211 is based on a relatively small animal population (4 and 3), the interpretation of these data must remain limited. It was noticeable that the most intense vacuolar lesion profile was found for the O100 isolate in which hypothalamus, thalamus and hippocampus were particularly triggered. It was somewhat similar to the lesion profile of O111 and O104, especially for the second passage profile (Fig. 1).

**Table 1:** Second passage transmission studies of CH1641 sheep isolate inoculated i.c. to Tg(OvPrP4) mice, compared to murine scrapie strains (C506M3, 87V, 79A and Chandler), “CH1641-like” natural isolates and other natural isolates, including BSE.

Prion sources into Tg(OvPrP4) mice	Mean survival periods at 1^st^ passage	*Attack rate*	Mean survival periods at 2^nd^ passage	*Attack rate*
***Ovine scrapie strain***				
CH1641	245+/−17 [26]	*12/12*	220+/−31	*12/12*
***Murine scrapie strains***				
C506M3	350 [21]	*4/8*	333+/−26	*12/12*
Chandler	450 [21]	*11/21*	396+/−100	*9/11*
79A	540 [21]	*6/15*	342+/−20	*12/12*
87V	460 [21]	*9/16*	258+/−44	*8/10*
***“CH1641-like” natural ovine isolates***				
TR316211	235+/−26 [26]	*8/9*	221+/−31	*8/11*
O104	248+/−50 [26]	*10/10*	238+/−29	*13/13*
O100	364+/−61 [26]	*12/12*	Not available	
***Other natural isolates***				
O111	296+/−20 [26]	*10/11*	399+/−124	*8/10*
Cattle BSE	421+/−48	*10/10*	354+/−48	*10/10*

Incubation periods are expressed in days as the mean +/− standard error (SE) of the mean. The incubation periods of first passage experiments already reported elsewhere (data from [6], [18], [21]) are recalled in order to facilitate interpretation of the second passage data. In the case of murine scrapie strain in accordance with [21], only approximations of mean incubation periods in days are reported. Attack rates are given to indicate transmission efficacy (number of PrPsc(IHC)/PrPres(WB) positive mice/total number of inoculated mice). Compared to first passage experiment, the unexpected prolonged survival of ovine scrapie O111 with large SE may result from the PrPc expression level in this mouse model as described in atypical scrapie transmission studies published previously [33].

**Figure 2:**
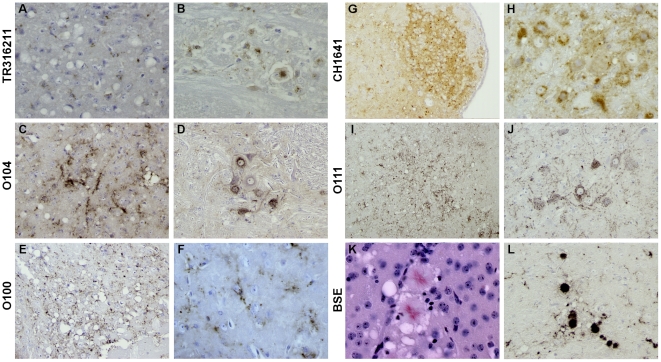
Illustrations of the main type of PrPd deposition as revealed by IHC using SAF84 mAb detected in the brain of TgOvPrP4 mice infected with either “CH1641-like” French scrapie isolates (TR316211(A, B), O104 (C, D) , O100 (E, F)), CH1641 sheep scrapie (G, H), or other natural isolates (sheep scrapie O111 (I, J) and cattle BSE (K, L)). The main type of PrP^d^ deposition was granular (A–H), and within the cytoplasm of neuronal cell bodies ((B, D, F, H, J), except for the BSE strain for which typical deposition as florid plaques was systematically and predominantly observed (K, L). The amyloid nature of these florid plaques was revealed by examining its birefringence property under polarized light on a section stained with Congo red (K).

The PrP^d^ brain mapping was comparable for these biochemically-defined cases of “CH1641-like” sheep scrapie - especially in the caudal areas - but the intensity of pathological PrP accumulation varied between isolates. The second passage experiments of TR316211 and O104 isolates led to notably greater PrP^d^ accumulation, revealing close similarities with PrP^d^ brain mapping. The O111 isolate, defined as biochemically different from the “CH1641-like” sheep scrapie group, caused less PrP^d^ deposition, especially in the rostral parts of the brain. The cortex, septum, caudate-putamen, amygdala and hippocampus gray matter sites thus remained devoid of any PrP^d^ accumulation unlike the “CH1641-like” scrapie group. It should be noted that the types of PrP^d^ deposition were similar (Fig. 2 A–F) in the “CH1641-like” sheep scrapie group (Fig. 2 A–F) and the O111 isolate (Fig. 2 I and J). PrP^d^ deposition was fine granular and sometimes intraneuronal (Fig. 2 B, D, F, J) but never in the form of plaques as observed with BSE (Fig. 2 L).

### Second passage experiments of BSE and scrapie murine-adapted strains

**Figure 3:**
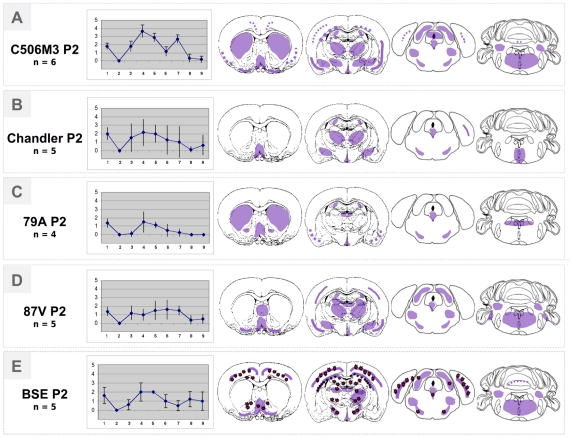
Lesion profiles and PrP^d^ mapping (schematic representation of PrP^d^ distribution in violet within 4 brain levels analyzed) observed in the brain of Tg(OvPrP4) mice (n = 4 to 6) infected with either C506M3, Chandler, 79A, 87V or BSE strains on second passage. Red dots symbolize amyloid florid plaques typical of the BSE infectious agent detected in this transgenic mouse model.

**Figure 4:**
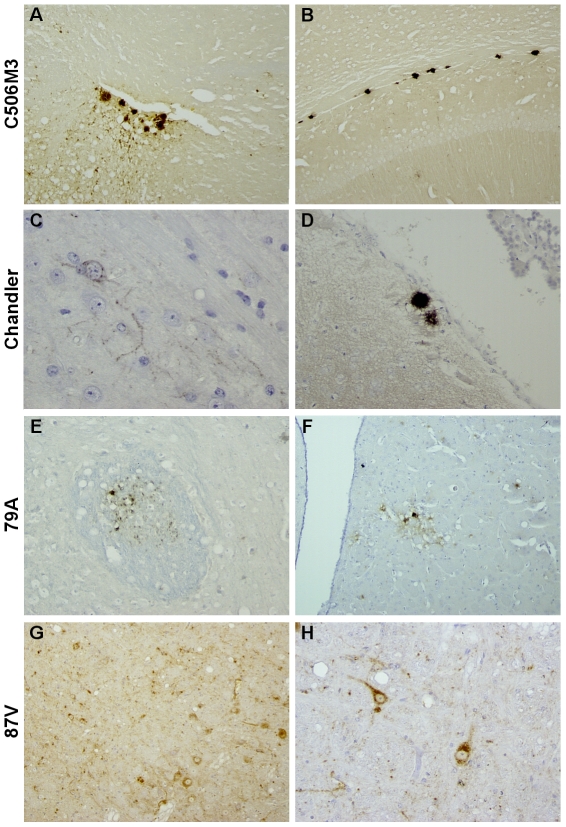
Illustrations of the main type of PrPd deposition as revealed by IHC using SAF84 mAb detected in the brain of TgOvPrP4 mice infected with either C506M3 (A, B), Chandler (C, D), 79A (E, F), or 87V (G, H) scrapie strains. PrPd was detected as amyloid plaques (A, B, D), fine granular linear and intraneuronal deposits (C, F, G, H). In the case of the 79A strain granular PrP^d^ deposition was seen within the white matter (E).

Table 1 and Figures 3 and 4 give a detailed comparison of BSE and scrapie murine-adapted strains (C506M3, Chandler, 79A and 87V strains) transmitted in a second passage experiment with TgOvPrP4 mice. Table 1 reports the incubation periods and attack rates observed for each murine scrapie strain and reveals a noticeable decrease compared to first passage experiments already published and reviewed in Table 1 [21]. The smallest decrease was observed for the C506M3 and Chandler strains, whereas for the 79A and 87V strains, the mean incubation periods appeared to be drastically shorter than the first passage data. This resulted in the 87V strain becoming the fastest of all the strains (258 days post inoculation +/−44). The range of survival periods differed from the “CH1641-like” group. The histopathological strain characteristics summarized in Figure 3 confirmed the possibility of distinguishing the different strains on the basis of vacuolation and accumulation of PrP^d^ within the brain of each mouse analyzed. The C506M3 and Chandler strains caused the most severe vacuolar lesions, revealed by their lesion profiles (n = 6 and n = 5 respectively) (Fig. 3). The lesion profiles differed from each other and from those in the “CH1641–like” group. All these vacuolar lesions were associated with a different type of PrP^d^ deposition (Fig. 4 A, E, F, H), which in this case was a synaptic-like fine granular deposition (Fig. 4 C), often within neuronal cytoplasm (Fig. 4 C, G, H). Apart from these granular types of PrP^d^ deposition, PrP^d^ plaques were also seen (Fig. 2 K, L, and Fig. 4 A, B, D). Some were amyloid plaques, revealed by birefringence observed under polarized light after Congo red staining (Fig. 2 K). These amyloid plaques were observed only for the C506M3, Chandler and BSE strains (i.e. within the subcallosum region, the cortex or the thalamus Fig. 2 K, L and Fig. 4 A, B, D). As already reported in the BSE transmission study, PrP^d^ plaques were surrounded by a regular ring of vacuoles which, in this model, define the specific BSE histopathological attribute of florid plaques (Fig. 4 K, J). The amyloid nature of PrP^d^ deposition detected in the first passage experiment for 79A was no longer detected in these second passage experiments. The most remarkable observation, however, was the absence of PrP^d^ amyloid plaques as the main type of deposition for the 87V strain.

### PrP^res^ brain mapping using a differential PET-Blot analysis

**Figure 5:**
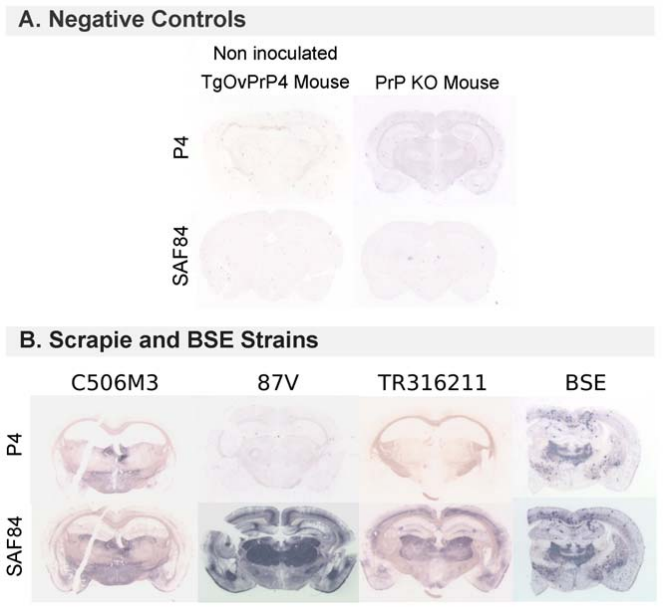
Epitopic PET-Blot analysis. Illustrations of comparative PrP^res^ detection with P4 and SAF84 mAb using a PET-blot analysis. A. The brains of a non inoculated TgOvPrP4 mouse and a PrP KO mouse were used as negative controls and showed no PrP^res^ deposition. B. The brains of a TgOvPrP4 mouse inoculated with C506M3, 87V or TR316211 or with BSE strains by the i.c. route. PrP^res^, observable as dark blue deposits on these membranes, was detectable in each case using SAF84 but not using P4 mAb. PrP^res^ revealed by this antibody was present in the C506M3 and BSE experiment and not in the second passage experiment of 87V or the TR316211 isolate. In C506M3- just as in BSE-infected brains, P4-labeled PrP^res^ accumulated in less specific brain areas and in smaller quantities than shorter PrP^res^ forms detected by the SAF84 prion antibody.

In an attempt to clarify the characteristics of this specific group of “CH1641-like” sheep scrapie isolates from a histopathological point of view, an initial set of experiments was performed using a differential PET-blot approach. Interestingly, by using two different monoclonal antibodies recognizing two different epitopes of the prion protein, it was possible to reproduce a distinction that had been previously observed biochemically between the “CH1641-like” sheep scrapie and another sheep isolate (O111) or mice-adapted scrapie strains derived from wild-type mice. Figure 5 illustrates our comparison of P4 mAb and SAF84 mAb labeling with the example of second passage experiments with positively labeled C506M3 and BSE compared to 87V and TR316211 unstained with P4 mAb. In an additional comparison to negative controls such as a non infected TgOvPrP4 mouse or a PrP KO mouse, (Fig. 5 A), the use of P4 mAb did not detect any PrP^res^ in 87V-infected transgenic mice nor in CH1641-, TR316211-, O104- or O100-infected mice (Fig. 5B). However, some mice from the O100 experiment contained PrP^res^ molecules, detected using P4. This result implies individual variations within the experimental group. Interestingly, in the group of mice positively labeled using the P4 antibody, such as BSE, C506M3 or 79A, the presence of PrP^res^ was revealed by P4 mAb to a much lesser degree than by SAF84 mAb (Fig. 5).

## Discussion

In previous publications on CH1641 scrapie source transmission studies, only biochemical data and no histopathological data were provided. In these studies, CH1641 transmission data were analyzed in comparison to the natural “CH1641-like” group (TR316211, O100 and O104) and not the “CH1641-like” isolate (O111), in addition to classical murine scrapie strains and BSE strains (defined according to their origin and biochemical criteria) [6], [18]. Importantly, these biochemical criteria implied that the 87V scrapie strain and BSE shared a low apparent molecular mass of unglycosylated PrPres which did not react very much to the P4 antibody and which, as far as PrPres cleavage is concerned, appeared comparable to the situation in some (O100 and O104) or all (TR316211 and CH1641) of the TgOvPrP4 mice infected with “CH1641-like” sheep TSE samples. In contrast, TgOvPrP4 mice infected with the C506M3, Chandler or 79A scrapie strains had higher apparent molecular masses of unglycosylated PrPres combined with intense PrPres labeling by P4 antibody. In the present histopathological study, the vacuolar lesion profiles and PrP^d^ brain mapping data for the natural “CH1641-like” group showed more similarities than divergences, which may be attributed at least in part to the intensity of PrP^d^ accumulation, as suggested by the comparative data between 1^st^ and 2^nd^ passage experiments with TR316211 and O104 isolates. Nonetheless, the vacuolar lesion profiles for CH1641 and TR316211 appeared similar to each other but clearly different from the O104 and O100 group. It is therefore possible to group strains in accordance with the biochemical study grouping [6], [18]. However, these results are not easy to interpret, especially because there were too few mice available to establish the TR316211 lesion profile.

The data on the BSE and scrapie murine-adapted strains (C506M3, Chandler, 79A and 87V strains) transmitted in a second passage experiment to TgOvPrP4 mice showed clear differences between strains in terms of survival periods, lesion profiles, PrP^d^ brain mappings and PrP^d^ deposition type. Ongoing third-passage experiments should provide some explanations for some of the unexpected evolutions in the histopathological data recorded, especially for the 79A and 87V strains. While waiting for the results of this third-passage experimental study, we have considered analyzing the present data further by developing a differential PET-blot analysis. In an attempt to find ways of unifying the biochemical and histopathological data on strain typing, we decided to pursue the histopathological analysis of the brains of TgOvPrP4 mice infected with these scrapie sources. An initial set of experimental data underwent an epitopic PET blot analysis, developed on the basis of the comparative use of SAF84 and P4 mAbs that recognized two different epitopes of the prion protein. Initial findings showed that it is possible to reproduce a classification based on P4 reactivity. The BSE, C506M3, Chandler and 79A groups showed P4-detected PrP^res^ molecules, unlike the 87V, CH1641 and TR316211 group. Some mice from the O100 experiment had PrP^res^ molecules detected using P4, implying individual variations within the experimental group, an implication also suggested by the wide variations in lesion profiles consistent with biochemical analyses [18]. It should be noted that for all mice in this group positively labeled by P4, the PrP^res^ labeling was less intense than that detected using SAF84. All the regions that accumulated PrP^res^ detected using P4 were systematically SAF84–positive too, whereas not all the regions that accumulated PrP^res^ detected using SAF84 were positively labeled using P4 mAb. This may indicate that only specific brain regions or cell types may express the PrP^res^ molecules detected using P4, whereas more brain regions and cell types express PrP^res^ molecules detectable only with SAF84. We cannot exclude the possibility that the P4 antibody may be less sensitive. However, this observation was reproducible using another prion antibody recognizing the same epitopic region recognized by P4 (data not shown). To better characterize the type of cells concerned, a detailed differential immunohistochemical analysis of these specific regions will be necessary. This would actually be a suitable way of apprehending the biological origin of these different forms of PrP^d^ detected *in situ*. As observed in the sheep species, these different kinds of immunoreactivity probably reveal different levels of naturally-occurring enzymatic cleavage of the PrP^d^, which may depend on the strain of agent and the type of brain cell considered [27]. However, this approach would probably raise a new set of issues because the epitopic PET-Blot focuses on the results of the controlled enzymatic digestion of PrPd molecules using PK *in situ*, in other words the PrPres molecules themselves. It does not investigate the “natural truncation” of PrPd molecules that exists at cellular level. An epitopic PET Blot analysis is an original way of obtaining an approximation *in situ* of the length of the PrPres molecules accumulated in the brain of mice experimentally infected with different prion strains. The advantage is that this method can be used to characterize a variety of PrPres molecules—similar to detection by WB—directly *in situ* within the brain depending on the prion strains. Unfortunately, we cannot at this stage identify, of all the PrP^res^ molecules detected *in situ* using SAF84, which ones may be related to the additional PrP^res^ fragment (named PrP^res^ #2) described recently by our group [6]. This C-terminally cleaved PrP^res^ product detected using SAF84 mAb specifically in the “CH1641-like” isolates compared to 79A, Chandler and C506M3 strains and transmitted to TgOvPrP4 mice, helps differentiate these prion groups.

Many more experiments related to these biochemically-defined groups, together with the use of several other possible anti-prion antibodies, are already planned. Importantly, this complementary histopathological tool already provides data showing strain properties concordant with the biochemical characterization of this particular group of prion agents.

## Materials and Methods

### Ethics statement

All procedures were carried out in compliance with the guidelines laid down by the Regional (CREEA no. 98) and French (Decree 87–848) Ethical Committees and European Community Directive 86/609/EEC. Animal experiments were performed in the ANSES animal facilities which have the relevant approval to carry out animal work (A 69 387 0801) by licensed people working in the animal experiment unit (license numbers AB: 69 387 531, LL: 69 387 191).

### Scrapie agents

The different scrapie sources used are presented in Table 1. Some of them are derived from mouse-adapted strains (79A, 87V, C506M3 and Chandler) transmitted in a first passage experiment by the i.c. route to TgOvPrP4 mice, or sheep-passaged isolates (CH1641), as well as four natural French TSE isolates, three “CH1641-like” isolates (TR316211, O100 and O104) and one not CH1641-like (O111). Transmission studies and the molecular analyses of CH1641 and of other natural “CH1641-like” isolates transmitted to TgOvPrP4 ovine transgenic mice have already been described [6], [18], [28].

### Experimental design

Groups of 12 female TgOvPrP4 mice [29], 4 to 6 weeks old, were challenged intracranially with 20 µl of 10% (1^st^ passage) or 1% (2^nd^ passage) (wt/vol) brain homogenates in 5% glucose in distilled water. This transgenic mouse model (TgOvPrP4) overexpresses in the brain, between 1.5 and 6 times the levels of the PrPARQ ovine prion protein under the control of the neuron-specific enolase promoter [29]. A source of BSE agent was also transmitted to TgOvPrP4 mice, used as a control group for the CH1641 group. The mice were euthanized at the terminal stage of disease development.

### Histological examinations

On death, the mouse brains were removed and processed either for biochemical (frozen at −20°C) or histochemical (fixed in 4% buffered paraformaldehyde) detection of PrP^d^ following procedures published in detail elsewhere [21], [30]. Amyloid deposits and vacuolar lesions were examined on brain sections stained with Congo red [22] and hematoxylin-eosin (HE) respectively. Lesion profiles were established according to Fraser's lesion profile [31] by quantification using a computer-assisted method (Morpho Expert software, Explora Nova, La Rochelle, France) [32]. Brain slices were immunostained for PrP^d^ as previously described with pre-treatments designed to enhance PrP^d^ detection using SAF84 (SPI Bio, Massy, France) or P4 monoclonal antibody (R-Biopharm, St Didier au Mont d'Or, France) which maps to the regions _167_RPVDQY_172_ and _93_WGQGGSH_99_ of ovine PrP respectively [20], [21]. A peroxidase-labeled avidin-biotin complex (Vectastain Elite ABC, Vector Laboratories, Burlingame, CA) was used to amplify the signal.

To visualize *in situ* the resistant form of abnormal PrP (PrP^res^) after digestion with a high concentration of proteinase K (PK), the PET-blot method was used as previously described [26]. Briefly, 5-µm-thick paraffin sections were collected onto 0.45 µm pore nitrocellulose membranes (Biorad, Marne la Coquette, France). The membranes were dewaxed and dried at room temperature (RT). After wetting with TBST (10 mM Tris HCl, pH 7.8; 100 mM NaCl; 0.05% Tween 20), enzymatic digestion was performed using 250 µg/ml PK (Roche-Boehringer, Meylan, France) in a buffer made of 10 mM Tris HCl, pH 7.8, 100 mM NaCl, 0.1% Brij 35, for 8 hours at 55°C. Membranes were treated with guanidine isothiocyanate (3 M, 10 min), then thoroughly washed in TBST. Immunodetection was performed after pre-incubation in a blocking solution (skimmed milk diluted at 0.2% in TBST). The monoclonal antibody used was either SAF84 (1/2500) or P4 (1/1000) for one night at RT. A phosphatase alkaline coupled anti-mouse antibody (Clinisciences, Montrouge, France) was used as the secondary antibody (1/500, 37°C, 45 min). Before revelation, the pH was adjusted to 9 by incubating membranes in NTM buffer (100 mM Tris-HCl, pH 9.5, 100 mM NaCl, 50 mM MgCl_2_). Finally, NBT/BCIP (Clinisciences, Montrouge, France) was used to visualize the reaction product (dark blue deposits). PET-blot membranes were assessed using a stereo-microscope (Olympus, Rungis, France) linked to an image analysis workstation (Explora Nova, La Rochelle, France).
